# Supplementary material for: Empirical methods for controlling false positives and estimating confidence in ChIP-Seq peaks
Source: BMC Bioinformatics. 2008 Dec 5;9:523. doi: 10.1186/1471-2105-9-523 (PMC2628906; doi:10.1186/1471-2105-9-523)
Supplement: Additional File 1 — A variety of html documents from the USeq web site detailing the available applications, their best usage, output file type descriptions, command line menus, etc. [file 1471-2105-9-523-S1.zip › USeqUserGuides/JavaDoc.zip!/JavaDoc/util/bio/converters/package-summary.html]

util.bio.converters


|  |  |  |
| --- | --- | --- |
| **Overview** \| **Package** \| Class \| **Use** \| **Tree** \| **Deprecated** \| **Index** \| **Help** | |  |
| **PREV PACKAGE**   **NEXT PACKAGE** | **FRAMES**    **NO FRAMES**     **All Classes** |  |


---

## Package util.bio.converters

Bio format converters.

**See:**
  
          **Description**

| **Class Summary** | |
| --- | --- |
| **AlignedBaseCounter** | Counts and parses the bases in a multiple alignment where each string is broken into three sections like gatcc GGCCAATT cgatcca, ccat GGCCAATT cctttt, like the output from meme the central portion is aligned, the left and right flanks are not. |
| **GenomeFASTASplitter** | Splits a whole genome fasta into different files, also rewrites the fasta header to just the first word following the > |
| **PossumToGFF** | Converts a copied Possum output to GFF annotation, prints to screen, sorted by score. |
| **RepeatMaskerToGFF** | Converts a RepeatMasker output to GFF annotation. |

## Package util.bio.converters Description

Bio format converters.

---


|  |  |  |
| --- | --- | --- |
| **Overview** \| **Package** \| Class \| **Use** \| **Tree** \| **Deprecated** \| **Index** \| **Help** | |  |
| **PREV PACKAGE**   **NEXT PACKAGE** | **FRAMES**    **NO FRAMES**     **All Classes** |  |


---
